# Clinical inertia in basal insulin-treated patients with type 2 diabetes – Results from a retrospective database study in Japan (JDDM 43)

**DOI:** 10.1371/journal.pone.0198160

**Published:** 2018-09-18

**Authors:** Jo Satoh, Marc Andersen, Brian Bekker Hansen, Brian Larsen Thorsted, Deniz Tutkunkardas, Mette Zacho, Hiroshi Maegawa

**Affiliations:** 1 Tohoku Medical and Pharmaceutical University Wakabayashi Hospital, Sendai, Japan; 2 StatGroup ApS, Copenhagen, Denmark; 3 Global Market Access, Novo Nordisk A/S, Søborg, Denmark; 4 Novo Nordisk A/S, Søborg, Denmark; 5 Global Medical Affairs, Novo Nordisk A/S, Søborg, Denmark; 6 Novo Nordisk Pharma Ltd, Tokyo, Japan; 7 Shiga University of Medical Science, Ōtsu, Japan; Weill Cornell Medical College Qatar, QATAR

## Abstract

**Aims:**

This retrospective cohort study investigated whether clinical inertia, the failure to intensify treatment when required, exists in Japanese clinical practice, using the CoDiC^®^ database. How and when patients with type 2 diabetes treated with basal insulin received treatment intensification was also described.

**Materials and methods:**

Patients with type 2 diabetes who initiated basal insulin between 2004 and 2011 were eligible for inclusion. Patients with an HbA1c ≥7.0% (≥53.0 mmol/mol) after 180 days of basal insulin titration were eligible for intensification, and their treatment was followed for up to 1.5 years. Endpoints were time to intensification, changes in HbA1c, and insulin dose.

**Results:**

Overall, 2351 patients initiated basal insulin treatment (mean HbA1c 9.4% [79.2 mmol/mol]), and 1279 patients were eligible for treatment intensification (HbA1c ≥7.0% [≥53.0 mmol/mol]) after the 180-day titration period. During the 1.5-year follow-up period (beyond the 180-day titration period), 270 (21%) of these patients received treatment intensification. In patients receiving treatment intensification, mean HbA1c decreased from 8.6 to 8.2% (70.5 to 66.1 mmol/mol) at end of follow-up. Treatment was intensified using bolus insulin in 126 (47%) patients and with premixed insulin in 144 (53%) patients. The estimated probability of intensifying treatment during the 12 months after recording HbA1c ≥7.0% (≥53.0 mmol/mol) was 22.8%, and 27.5% after 17 months. Mean end-of-follow-up daily insulin dose was 35.11 units for basal–bolus compared with 20.70 units for premix therapy.

**Conclusions:**

This study suggests clinical inertia exists in basal insulin-treated patients with type 2 diabetes in Japan. Strategies are needed to increase the number of patients undergoing therapy intensification and to reduce the delay in intensification in Japan.

## Introduction

The progressive nature of type 2 diabetes (T2D) necessitates that patients intensify their treatment in order to maintain glycemic control [1]. As insulin is the most effective glucose-lowering therapy, most patients will eventually use an insulin-based regimen [2]. The treatment algorithms described in consensus guidelines recommend a step-wise approach to intensifying treatment. Following lifestyle and diet changes, patients usually start with oral antidiabetic drugs (OADs), then as their diabetes advances, a glucagon-like peptide-1 receptor agonist (GLP-1RA) and/or a basal insulin may be introduced as required [3, 4]. Treatment can then be further intensified, for example with meal-time (bolus) insulin. Guidelines from the American Diabetes Association/European Association for the Study of Diabetes also include GLP-1RAs as an intensification option at this stage (if not already being used) [3]. Intensification is recommended if glycated hemoglobin (HbA1c) remains above the glycemic target for 3 months, with a target of 7.0% recommended for most patients [3, 4]. Patients whose treatment is not intensified as recommended, and thus remain in poor glycemic control, are at a greater risk of long-term diabetes-related complications, such as retinopathy and nephropathy [5, 6]. Several studies have demonstrated that patients with T2D frequently do not reach glycemic target and their treatment intensification is often delayed, both in terms of initiating insulin and later intensifying insulin regimens–this is known as clinical inertia [7–10]. Clinical inertia is a global issue that has shown little improvement in prevalence over the last decade [8]. It is unsurprising, therefore, that a huge financial burden has been anticipated due to complications arising from uncontrolled T2D associated with clinical inertia [11]. Increasing awareness may be a key step in overcoming the failure to intensify therapy. It is, therefore, important to obtain local, real-world evidence to assess the extent of clinical inertia in different regions, so that strategies can be designed to improve clinical care and outcomes.

In 2010, Japan had the world’s eighth highest number of patients (aged 20–79 years) with diabetes [12], yet data on the existence of clinical inertia in patients with T2D in Japanese clinical practice are lacking. The aim of this retrospective cohort study was to investigate whether clinical inertia exists in Japanese clinical practice using data from the CoDiC^®^ (Computerized Diabetes Care) database of the Japan Diabetes Clinical Data Management Study Group (JDDM), and to describe how and when patients with T2D treated with basal insulin received treatment intensification.

## Materials and methods

The data for this retrospective cohort study were extracted from the CoDiC database, a large, anonymized, longitudinal, validated database containing patient-level clinical information including diagnoses, mortality, laboratory results and prescription data sourced from multiple diabetes institutions across Japan. CoDiC was developed by the JDDM to promote clinical research on diabetes. The database holds records for approximately 60,000 patients from 61 institutions across Japan and is updated annually [13]. All collected data were held at a central analytical center established by the JDDM, where patient data were anonymized prior to analysis. The JDDM ethics committee approved the study protocol and patients were informed of the data collection, as per the Guideline for Epidemiology Study in Japan requirements [14].

**Fig 1:**
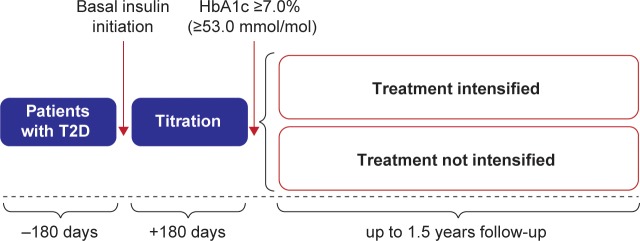
Study design. HbA1c, glycated hemoglobin; T2D, type 2 diabetes.

The study design is shown in Fig 1. Patients were included in the overall study population if they had a diagnosis of T2D based on the Japanese Diabetes Society criteria [15], were aged ≥18 years, and were initiated on basal insulin between 2004 and 2011. Patients were eligible for treatment intensification if they had at least 180 days of data from before basal insulin initiation, an HbA1c ≥7.0% (≥53.0 mmol/mol) after 180 days of basal insulin treatment, and up to 1.5 years of follow-up data. Patients were excluded if they had prior use of bolus or premixed insulin or GLP-1RA therapy or had gaps of more than 1 month without recording insulin dose. The 180-day cut-off after insulin initiation was used to allow for sufficient time for titration of basal insulin prior to initiating additional therapies.

An alternative HbA1c threshold of ≥8.0% (≥63.9 mmol/mol) after 180 days of basal insulin treatment was also used to complement the main HbA1c threshold (≥7.0% [≥53.0 mmol/mol]) for treatment intensification.

Patients visited their clinician every 1 to 2 months and received laboratory examinations. Clinical data were collected once per year for the CoDiC database, with prescription data, HbA1c, weight, body mass index (BMI) available as monthly aggregated data.

Endpoints were time to intensification, changes in HbA1c and insulin dose.

### Statistical methods

Characteristics of the study population were described for five cohorts with number (%) and mean (standard deviation [s.d.]). The five cohorts presented are 1. overall population (patients with T2D who initiated basal insulin); 2. patients who initiated basal insulin and were not eligible for treatment intensification (HbA1c <7.0% [<53.0 mmol/mol] after 180 days); 3. patients who initiated basal insulin and were eligible for treatment intensification (HbA1c ≥7.0% [≥53.0 mmol/mol] after 180 days); 4. patients with a HbA1c ≥7.0% (≥53.0 mmol/mol) who received treatment intensification during the follow-up period; and 5. patients with a HbA1c ≥7.0% (≥53.0 mmol/mol) who did not receive treatment intensification during the follow-up period.

A Kaplan–Meier estimator was applied to estimate the distribution of time from first time eligible for intensification to treatment intensification. Patients who did not intensify treatment were censored if HbA1c dropped below 7.0% (53.0 mmol/mol), or transferred out of the database in the case of death or if there were missing data. HbA1c and insulin dose are reported with mean (s.d.) at the specified time points.

Key results from patients with a HbA1c ≥8.0% (≥63.9 mmol/mol) after 180 days of basal insulin treatment are shown in addition to the results obtained using the main HbA1c threshold (≥7.0% [≥53.0 mmol/mol]) for treatment intensification.

## Results

### Population demographics and baseline characteristics

The clinical characteristics of the study population cohorts are summarized in Table 1. There were 2351 patients who initiated treatment with basal insulin. At the index date (when basal insulin was initiated), the study population were 64% male, had a mean age of 60.7 years, and a mean BMI of 24.5 kg/m^2^.

**Table 1:** Study population characteristics at index date (when basal insulin was initiated).

	Total (N = 2351)		Not eligible for intensification (HbA1c <7.0% [<53.0 mmol/mol]) (n = 1072)		Eligible for intensification (HbA1c ≥7.0% [≥53.0 mmol/mol]) (n = 1279)		Eligible for intensification (HbA1c ≥7.0% [≥53.0 mmol/mol]), intensified (n = 270)		Eligible for intensification (HbA1c ≥7.0% [≥53.0 mmol/mol]), not intensified (n = 1009)	
	N	Mean (s.d.)	n	Mean (s.d.)	n	Mean (s.d.)	n	Mean (s.d.)	n	Mean (s.d.)
**Age, years**	2351	60.7 (12.9)	1072	59.7 (13.5)	1279	61.4 (12.4)	270	58.6 (12.3)	1009	62.2 (12.3)
**Gender, male %**	2351	64%	1072	64%	1279	64%	270	64%	1009	64%
**Duration of diabetes, years**	2338	3.9 (5.3)	1063	3.5 (5.0)	1275	4.3 (5.6)	269	3.7 (4.4)	1006	4.5 (5.8)
**BMI, kg/m**^**2**^	2065	24.5 (4.4)	927	24.5 (4.6)	1138	24.5 (4.2)	255	25.1 (4.3)	883	24.3 (4.1)
**Body weight, kg**	2131	63.9 (14.6)	968	64.5 (15.5)	1163	63.4 (13.9)	260	65.6 (13.8)	903	62.7 (13.8)
**HbA1c, %**	2230	9.4 (1.9)	1002	9.5 (2.1)	1228	9.4 (1.8)	266	9.5 (1.7)	962	9.4 (1.8)
**No OADs, %**	672	28.6%	363	33.9%	309	24.2%	36	13.3%	273	27.1%
**One OAD, %**	536	22.8%	243	22.7%	293	22.9%	53	19.6%	240	23.8%
**At least two OADs, %**	1143	48.6%	466	43.5%	677	52.9%	181	67.0%	496	49.2%
**Basal insulin, units**	2351	9.8 (7.3)	1072	9.9 (7.4)	1279	9.8 (7.3)	270	8.7 (7.1)	1009	10.1 (7.4)
**Basal insulin, units/kg**	2131	0.16 (0.12)	968	0.16 (0.12)	1163	0.16 (0.12)	260	0.14 (0.10)	903	0.17 (0.12)

BMI, body mass index; HbA1c, glycated hemoglobin; OAD, oral antidiabetic drug; s.d., standard deviation.

In total, 1279 patients were eligible for treatment intensification (HbA1c ≥7.0% [≥53.0 mmol/mol]) after the 180-day titration period, and hence included in the study. In the patients eligible for intensification, the gender distribution and mean BMI were similar, and mean age was 61.4 years (Table 1). BMI was slightly below 25 kg/m^2^, which is considered to be the threshold for obesity in Japan, and was considered representative of T2D population in Japan. When using the threshold of HbA1c ≥8.0% (≥63.9 mmol/mol), a total of 930 patients were eligible for treatment intensification after the 180-day titration period.

Of the patients eligible for intensification (HbA1c ≥7.0% [≥53.0 mmol/mol]), the subgroup who intensified with basal–bolus insulin had a higher proportion of men, were younger, had a lower duration of diabetes, and had higher body weight and BMI at the index date compared with the subgroup who intensified with premix insulin (**S1 Table**).

### Glycemic control

**Fig 2:**
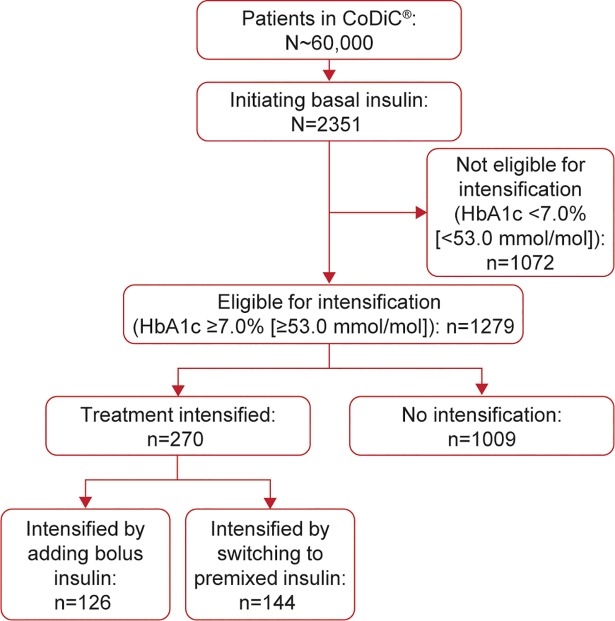
Patient disposition. CoDiC Computerized Diabetes Care database; HbA1c, glycated hemoglobin.

**Fig 3:**
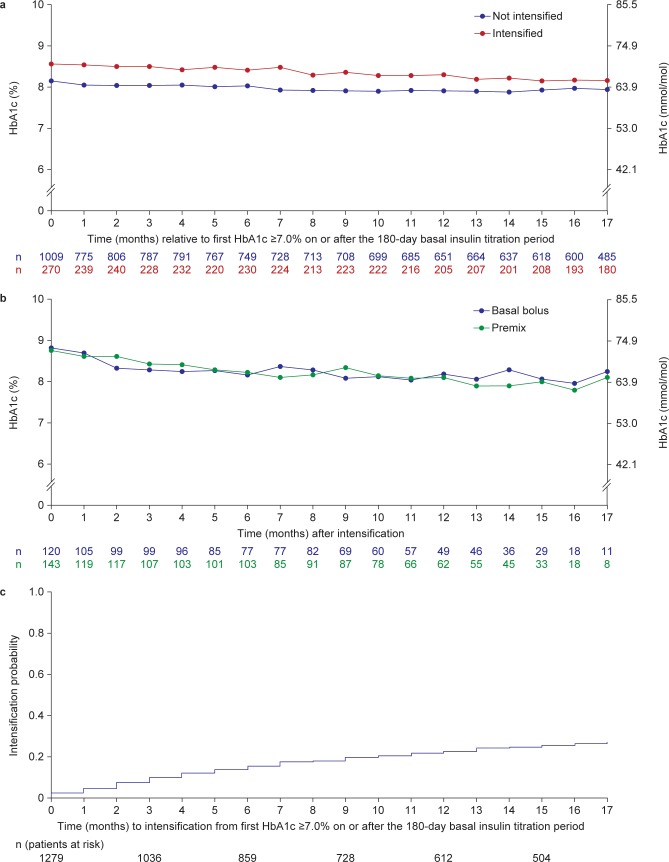
**a. Mean HbA1c over time relative to first HbA1c ≥7.0% (≥53.0 mmol/mol) on or after the 180-day basal insulin titration period, stratified by intensification status; b. Mean HbA1c over time after treatment intensification; c. Time to treatment intensification after HbA1c ≥7.0% (≥53.0 mmol/mol).** Fig 3A and 3B: n, number of patients contributing to each data point. HbA1c, glycated hemoglobin.

Mean (s.d.) HbA1c was 9.4% (1.9) [79.2 mmol/mol] in the 2351 patients initiating basal insulin. HbA1c ≥7.0% (≥53.0 mmol/mol) was reported in 1279 (54%) of these patients after the 180-day titration period, making them eligible for intensification. In the population eligible for intensification, the mean (s.d.) HbA1c was 9.4% (1.8) [79.2 mmol/mol] at basal initiation. During the 1.5-year follow-up period (beyond the 180-day titration period), 270 (21%) of these patients with HbA1c ≥7.0% (≥53.0 mmol/mol) received treatment intensification (Fig 2), and their mean (s.d.) HbA1c was 9.5% (1.7) [80.3 mmol/mol] prior to basal insulin initiation (Table 1). Treatment was intensified using bolus insulin in 126 (47%) patients and with premixed insulin in 144 (53%) patients. No patients had their treatment intensified with a GLP-1RA. Fig 3A shows the mean HbA1c for patients eligible for intensification split by patients who later intensified compared with patients who did not intensity treatment. For those who did not intensify, mean (s.d.) HbA1c decreased from 8.2% (1.2) [66.1 mmol/mol] at the time point eligible for intensification to 7.9% (1.2) [62.8 mmol/mol] after 17 months (beyond the 180-day titration period; Fig 3A). However, for those who later intensified, mean (s.d.) HbA1c decreased from 8.6% (1.2) [70.5 mmol/mol] at the time point eligible for intensification to 8.2% (1.3) [66.1 mmol/mol] after 17 months (beyond the 180-day titration period). Additionally for those who intensified, mean (s.d.) HbA1c at the time of intensification and 6 and 12 months later, was 8.8% (1.2) [72.6 mmol/mol], 8.2% (1.2) [66.1 mmol/mol], and 8.1% (1.3) [65.0 mmol/mol], respectively. Mean HbA1c over time after intensification with basal–bolus or premix insulin is shown in Fig 3B.

In the population eligible for intensification with a HbA1c ≥8.0% (≥63.9 mmol/mol) after the 180-day titration period, the mean (s.d.) HbA1c was 9.8% (1.8) (83.6 mmol/mol) at basal insulin initiation. A total of 231 (25%) of the 930 patients with HbA1c ≥8.0% (≥63.9 mmol/mol) after 180 days received treatment intensification during the 1.5-year follow-up period. Treatment was intensified using bolus insulin in 105 (45%) patients and with premix insulin in 126 (55%) patients.

The estimated probability of intensifying treatment during the 12 months after recording HbA1c ≥7.0% (≥53.0 mmol/mol; beyond the initial 180-day titration period) was 22.8%, and after 17 months was 27.5% (Fig 3C). The estimated probability of intensifying treatment during the 12 months after recording HbA1c ≥8.0% (≥63.9 mmol/mol) after the 180-day titration period was 28.1%, and 34.3% after 17 months.

### Insulin dose

**Fig 4:**
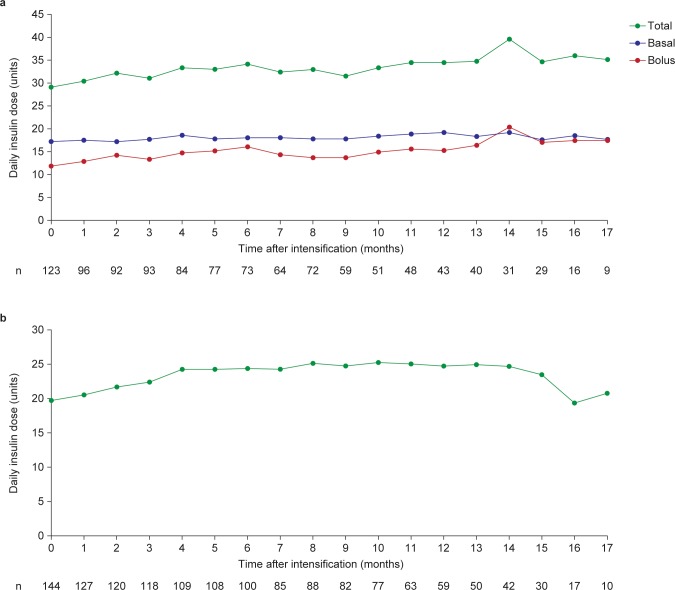
**Daily insulin dose after intensification by insulin regimen a. basal–bolus insulin b. premix insulin. Data are observed mean based on the number of patients on a particular regimen at a given time point.** n, number of patients with data for that time point. Patients who changed from basal–bolus insulin to premixed insulin, or vice versa, during the study contribute data to both panels of the figure.

At the time of initiation, the mean (s.d.) basal insulin dose was 9.8 units (7.3) [0.16 units/kg (0.12)] in patients eligible for intensification (HbA1c ≥7.0% [79.2 mmol/mol]). In patients eligible for intensification whose treatment was intensified, the mean (s.d.) basal insulin dose prior to intensification was 16 units (9.3; n = 248) [0.24 units/kg (0.14) (n = 230)]. Fig 4 shows mean insulin dose relative to intensification, stratified by insulin regimen. After treatment intensification, the total daily insulin dose in patients intensified with basal–bolus therapy, and those with premixed insulin therapy, both increased, which corresponded with the decreasing HbA1c in Fig 3B. The mean end-of-follow-up total dose was higher in patients intensified with basal–bolus therapy compared with the premix therapy (35.11 vs. 20.70 units, respectively; Fig 4).

## Discussion

It is estimated that approximately 7.2 million people in Japan have diabetes [16] and the importance of controlling patients’ glycemia to avoid diabetic complications is well established [5, 6]. This analysis of real-world data sought to investigate whether clinical inertia is apparent in patients with T2D in Japan. The CoDiC database contains records of patients treated by diabetes specialists in clinics, and as most patients in Japan are treated in a clinic rather than a hospital setting, it is considered representative of the diabetes population in Japan. This study found evidence of clinical inertia in Japanese clinical practice: of the patients with an HbA1c ≥7.0% (≥53.0 mmol/mol) after the 180-day titration period, the estimated probability of intensification by the end of follow-up was just 27.5%. There was a slightly higher probability of intensification when a HbA1c threshold of ≥8.0% (≥63.9 mmol/mol) after the 180-day titration period was assessed, with intensification taking place in 34.3% of patients.

The characteristics of the intensified population (HbA1c ≥7.0% [≥53.0 mmol/mol]) compared with the not intensified population were similar, but patients who were not intensified were on average older (mean age 62.2 vs. 58.6 years) and had a longer diabetes duration (mean 4.5 vs. 3.7 years) compared with those who were intensified, so these factors may have influenced the physician’s decision on whether or not to intensify treatment. Furthermore, more of the patients who were intensified (67%) were already on ≥2 OADs compared with those who were not intensified (49%). Although not measured in this study, the differences in age between populations may potentially be due to a higher number of diabetes-related comorbidities in patients who did not intensify [7]. The use of less stringent glycemic targets is recommended for patients with important comorbidities and long disease duration [2, 17]. The higher proportion of patients without concomitant OADs (27.1%) in the not intensified population compared with those who intensified (13.3%) suggests the use of such individualized targets. Furthermore, fewer of the patients who did not intensify were on ≥2 OADs (49%) compared with those who were intensified (67%). Therefore, as complications and comorbidities worsen with diabetes duration, the higher age of the not intensified population could be viewed as a correlate of the presence, or increased risk, of diabetes-related comorbidites.

Approximately half of patients with HbA1c ≥7.0% (≥53.0 mmol/mol) after the 180-day titration period who intensified treatment did so with bolus insulin and the other half with premix insulin. No patients intensified treatment with a GLP-1RA, this is likely to be because combined use of insulin and GLP-1RA were not permitted in Japan by the study end date of 2013. It is possible that some patients had their treatment intensified with an additional OAD, such as dipeptidyl peptidase-4 (DPP-4) inhibitors, which were introduced in Japan in 2009 and permitted for use in combination with insulin from 2011, but these data were not recorded in the database.

In patients with HbA1c ≥8.0% [≥63.9 mmol/mol] after the 180-day titration period, treatment was intensified with premix insulin in a higher proportion of patients than were intensified with bolus insulins.

Taking into consideration the recommendations of the consensus guidelines, the mean HbA1c in this study was high prior to insulin initiation (9.4% [79.2 mmol/mol]), especially compared with that reported for the overall T2D cohort in the CoDiC database (irrespective of the treatment received), in which mean HbA1c improved from 7.41% (57.5 mmol/mol) in 2001 to 7.06% (53.7 mmol/mol) in 2012 [18]. However, the poor level of glycemic control prior to insulin initiation is consistent with other studies, such as the Diabetes Attitudes Wishes and Needs (DAWN) Japan study, which also reported a mean HbA1c of 9.4% (79.2 mmol/mol) prior to insulin initiation [19], and the global SOLVE study, where pre-insulin HbA1c was 8.9% (73.8 mmol/mol) [20].

In the current study, in patients whose insulin treatment was intensified, mean HbA1c was 8.8% (72.7 mmol/mol) in those with HbA1c ≥7.0 (≥53.0 mmol/mol) after 180 days. In comparison, mean HbA1c was 7.5% (58.5 mmol/mol) in the SOLVE study [21] and 7.6% (59.6 mmol/mol) in the PREDICTIVE study [22], both 14 weeks after insulin initiation. The increase in basal insulin dose from initiation to prior to intensification was small in this study, suggesting that many patients, or their physicians, did not uptitrate their basal insulin sufficiently.

The results of this study are supported by the similar findings from the UK Clinical Practice Research Datalink, where only 30.9% of eligible patients (with HbA1c ≥7.5% [≥58 mmol/mol] >6 months after starting basal insulin) had their treatment intensified when followed for up to 10 years [7] compared with an estimated probability of intensification of 27.5% in patients with HbA1c ≥7.0 (≥53.0 mmol/mol) after 180 days, and 34.3% in patients with HbA1c ≥8.0% (≥63.9 mmol/mol) after 180 days in this study after up to 2 years’ follow-up. The higher estimated probability of intensification in patients with HbA1c ≥8.0% (≥63.9 mmol/mol) versus HbA1c ≥7.0 (≥53.0 mmol/mol) after 180 days suggests that treatment is more likely to be intensified for patients who have worse glycemic control. The mean HbA1c at intensification in eligible patients (HbA1c ≥7.5% [58 mmol/mol]) was higher in the UK-based study (9.8% [83.6 mmol/mol]) [7] compared with patients with HbA1c ≥7.0 (≥53.0 mmol/mol) after 180 days in this study (8.8% [72.7 mmol/mol]), possibly reflecting differences in clinical practice between the UK and Japan. Additionally, a previous study of patients treated with OADs from the CoDiC database showed that many of the patients who initiated basal insulin did not achieve glycemic control, and better control was observed in those who treatment was intensified with premixed or basal–bolus insulin [23]. The authors of that report suggested physicians should be aware that not all patients achieve glycemic target with basal insulin and the introduction of treatment regimens targeting prandial glucose should be considered (including both GLP-1RA and meal-time insulin) [23]. Of the patients with HbA1c ≥7.0% (≥53.0 mmol/mol) after 180 days in the present study, mean HbA1c decreased within the first 6 months following treatment intensification (with either bolus or premixed insulin) and continued to decrease steadily thereafter, falling from 8.8% (72.7 mmol/mol) at intensification to 8.1% (65.0 mmol/mol) after 12 months. An important consideration for both patients and physicians is the success of basal insulin intensification is dependent on sufficient dose titration.

The reasons for clinical inertia are multifactorial, but it has been suggested that improved communication between patients and physicians, acceptance of responsibility on both parts, and more resources to give healthcare professionals time to discuss treatments and diabetes management with patients would help reduce inertia [9, 24]. Several studies have shown patients and physicians perceive insulin regimens as a burden and difficult for patients to adhere to, with patients reporting they frequently miss or mistime their insulin doses [19, 25–27].

This study was not without limitations. The eligibility criteria for treatment intensification used in this study (HbA1c ≥7.0% [≥53.0 mmol/mol] after 180 days) did not include additional factors such as hypoglycemia and body weight, which could be considered if treatment goals are to be individualized. However, use of an additional HbA1c threshold (HbA1c ≥8.0% [≥63.9 mmol/mol] after 180 days) in this study provided insights into treatment intensification for patients with poor glycemic control after basal insulin initiation. Hypoglycemia, weight gain and patient acceptance of more complex treatment regimens may have influenced the frequency of or time to intensification, as well as the relatively low insulin doses used in the present study. However, these issues could not be addressed in the present study, as information regarding hypoglycemia and patient-reported outcomes were not captured in the CoDiC database. The limitations of the study also include the short duration of follow-up of up to 1.5 years. There may also be some bias in the characteristics of the patients included in the database as those treated in a hospital setting are not included. The CoDiC database contains clinical data on a large number of patients reflecting a real-life T2D population encountered in Japan. Data were input by members of the JDDM and physicians from specialized diabetes institutions, using JDDM guidelines, increasing the reliability of the data.

In conclusion, this retrospective cohort study of the CoDiC database investigated whether clinical inertia is apparent with respect to basal insulin intensification in patients with T2D in Japanese clinical practice. Only 21% of patients that had initiated basal insulin treatment and were eligible for treatment intensification (HbA1c ≥7.0% [≥53.0 mmol/mol]) after the 180-day titration period received treatment intensification during the 1.5 year follow-up period. The estimated probability of intensifying treatment during the 12 months after recording HbA1c ≥7.0% (≥53.0 mmol/mol) was 22.8%, and 27.5% after 17 months. This study has identified that clinical inertia is prevalent in basal insulin-treated patients with T2D in Japan. Strategies are needed to increase the number of patients undergoing therapy intensification and to reduce the delay in intensification.

## Supporting information

S1 Table Study population characteristics at index date (when basal insulin was initiated) for patients eligible for intensification (HbA1c ≥7.0% [≥53.0 mmol/mol]), and intensified, according to intensification treatment. BMI, body mass index; HbA1c, glycated hemoglobin; s.d., standard deviation.
(DOCX)

## References

[1] Fonseca VA. Defining and characterizing the progression of type 2 diabetes. Diabetes Care. 2009; 32 Suppl 2:S151–156. 10.2337/dc09-S301 19875543 PMC2811457

[2] Inzucchi SE, Bergenstal RM, Buse JB, Diamant M, Ferrannini E, Nauck M, et al Management of hyperglycemia in type 2 diabetes, 2015: a patient-centered approach: update to a position statement of the American Diabetes Association and the European Association for the Study of Diabetes. Diabetes Care. 2015; 38(1):140–149. 10.2337/dc14-2441 25538310

[3] American Diabetes Association. American Diabetes Association. Standard of Medical Care in Diabetes—2017. Diabetes Care. 2017; 40 (Suppl 1):1–142.

[4] Japan Diabetes Society. Evidence-based practice guideline for the treatment for diabetes in Japan 2013 [February 2017]. Available from: http://www.jds.or.jp/modules/en/index.php?content_id=27.

[5] Holman RR, Paul SK, Bethel MA, Matthews DR, Neil HA. 10-year follow-up of intensive glucose control in type 2 diabetes. The New England journal of medicine. 2008; 359(15):1577–1589. 10.1056/NEJMoa0806470 18784090

[6] Stratton IM, Adler AI, Neil HA, Matthews DR, Manley SE, Cull CA, et al Association of glycaemia with macrovascular and microvascular complications of type 2 diabetes (UKPDS 35): prospective observational study. BMJ (Clinical research ed). 2000; 321(7258):405–412. 10938048 10.1136/bmj.321.7258.405 PMC27454

[7] Khunti K, Nikolajsen A, Thorsted BL, Andersen M, Davies MJ, Paul SK. Clinical inertia with regard to intensifying therapy in people with type 2 diabetes treated with basal insulin. Diabetes Obes Metab. 2016; 18(4):401–409. 10.1111/dom.12626 26743666 PMC5067688

[8] Khunti K, Wolden ML, Thorsted BL, Andersen M, Davies MJ. Clinical inertia in people with type 2 diabetes: a retrospective cohort study of more than 80,000 people. Diabetes Care. 2013; 36(11):3411–3417. 10.2337/dc13-0331 23877982 PMC3816889

[9] Strain WD, Cos X, Hirst M, Vencio S, Mohan V, Voko Z, et al Time to do more: addressing clinical inertia in the management of type 2 diabetes mellitus. Diabetes Res Clin Pract. 2014; 105(3):302–312. 10.1016/j.diabres.2014.05.005 24956964

[10] Yam FK, Adams AG, Divine H, Steinke D, Jones MD. Clinical inertia in type 2 diabetes: A retrospective analysis of pharmacist-managed diabetes care vs. usual medical care. Pharmacy practice. 2013; 11(4):203–210. 24367460 10.4321/s1886-36552013000400005 PMC3869636

[11] Strain WD, Bluher M, Paldanius P. Clinical inertia in individualising care for diabetes: is there time to do more in type 2 diabetes? Diabetes Ther. 2014; 5(2):347–354. 10.1007/s13300-014-0077-8 25113408 PMC4269638

[12] Shaw JE, Sicree RA, Zimmet PZ. Global estimates of the prevalence of diabetes for 2010 and 2030. Diabetes Res Clin Pract. 2010; 87(1):4–14. 10.1016/j.diabres.2009.10.007 19896746

[13] Kobayashi M, Yamazaki K, Hirao K, Oishi M, Kanatsuka A, Yamauchi M, et al The status of diabetes control and antidiabetic drug therapy in Japan—a cross-sectional survey of 17,000 patients with diabetes mellitus (JDDM 1). Diabetes Res Clin Pract. 2006; 73(2):198–204. 10.1016/j.diabres.2006.01.013 16621117

[14] The Ministry of Health, Labor and Welfare. Guideline for Epidemiology Study in Japan. 2003:June 2003.

[15] Kuzuya T, Nakagawa S, Satoh J, Kanazawa Y, Iwamoto Y, Kobayashi M, et al Report of the Committee on the classification and diagnostic criteria of diabetes mellitus. Diabetes Res Clin Pract. 2002; 55(1):65–85. 11755481 10.1016/s0168-8227(01)00365-5

[16] International Diabetes Federation. International Diabetes Federation (IDF) Diabetes Atlas– 7th Edition 2015 [February 2017]. Available from: http://www.diabetesatlas.org/across-the-globe.html.

[17] Action to Control Cardiovascular Risk in Diabetes Study G, Gerstein HC, Miller ME, Byington RP, Goff DC Jr., Bigger JT, et al Effects of intensive glucose lowering in type 2 diabetes. The New England journal of medicine. 2008; 358(24):2545–2559. 10.1056/NEJMoa0802743 18539917 PMC4551392

[18] Japan Diabetes Clinical Data Management Study Group. Basic Summary Data 2013 [February 2017]. Available from: http://jddm.jp/data/index.html.

[19] Yoshioka N, Ishii H, Tajima N, Iwamoto Y, group DJ. Differences in physician and patient perceptions about insulin therapy for management of type 2 diabetes: the DAWN Japan study. Curr Med Res Opin. 2014; 30(2):177–183. 10.1185/03007995.2013.855187 24128339

[20] Khunti K, Damci T, Meneghini L, Pan CY, Yale JF, Group SS. Study of Once Daily Levemir (SOLVE): insights into the timing of insulin initiation in people with poorly controlled type 2 diabetes in routine clinical practice. Diabetes Obes Metab. 2012; 14(7):654–661. 10.1111/j.1463-1326.2012.01602.x 22443213

[21] Khunti K, Caputo S, Damci T, Dzida GJ, Ji Q, Kaiser M, et al The safety and efficacy of adding once-daily insulin detemir to oral hypoglycaemic agents in patients with type 2 diabetes in a clinical practice setting in 10 countries. Diabetes Obes Metab. 2012; 14(12):1129–1136. 10.1111/j.1463-1326.2012.01665.x 22830956

[22] Dornhorst A, Luddeke HJ, Sreenan S, Kozlovski P, Hansen JB, Looij BJ, et al Insulin detemir improves glycaemic control without weight gain in insulin-naive patients with type 2 diabetes: subgroup analysis from the PREDICTIVE study. Int J Clin Pract. 2008; 62(4):659–665. 10.1111/j.1742-1241.2008.01715.x 18324957

[23] Kanatsuka A, Sato Y, Kawai K, Hirao K, Kobayashi M, Kashiwagi A, et al Evaluation of insulin regimens as an effective option for glycemic control in patients with type 2 diabetes: A propensity score-matched cohort study across Japan (JDDM31). J Diabetes Investig. 2014; 5(5):539–547. 10.1111/jdi.12194 25411622 PMC4188112

[24] Zafar A, Stone MA, Davies MJ, Khunti K. Acknowledging and allocating responsibility for clinical inertia in the management of Type 2 diabetes in primary care: a qualitative study. Diabet Med. 2015; 32(3):407–413. 10.1111/dme.12592 25251768

[25] Atsumi Y, Brod M, Barnett AH, Peyrot M. GAPP2™: Global survey finds that a quarter of Japanese type 2 diabetes downplay insulin nonadherence to their healthcare professional. Diabetologia. 2012; 55 (Suppl 1):S397.

[26] Brod M, Rana A, Barnett AH. Adherence patterns in patients with type 2 diabetes on basal insulin analogues: missed, mistimed and reduced doses. Curr Med Res Opin. 2012; 28(12):1933–1946. 10.1185/03007995.2012.743458 23150949

[27] Peyrot M, Barnett AH, Meneghini LF, Schumm-Draeger PM. Insulin adherence behaviours and barriers in the multinational Global Attitudes of Patients and Physicians in Insulin Therapy study. Diabet Med. 2012; 29(5):682–689. 10.1111/j.1464-5491.2012.03605.x 22313123 PMC3433794

