# Supplementary material for: Clinical inertia in basal insulin-treated patients with type 2 diabetes – Results from a retrospective database study in Japan (JDDM 43)
Source: PLoS One. 2018 Sep 18;13(9):e0198160. doi: 10.1371/journal.pone.0198160 (PMC6143196; doi:10.1371/journal.pone.0198160)
Supplement: S1 Table — BMI, body mass index; HbA1c, glycated hemoglobin; s.d., standard deviation. (DOCX) [file pone.0198160.s001.docx]

**Supporting Information**

**S1 Table**. **Study population characteristics at index date (when basal insulin was initiated) for patients eligible for intensification (HbA1c ≥7.0% [≥53.0 mmol/mol]), and intensified, according to intensification treatment**

|  | **Basal bolus**  **(N = 126)** | | **Premix**  **(N = 144)** | |
| --- | --- | --- | --- | --- |
|  | **n** | **Mean (s.d.)** | **n** | **Mean (s.d.)** |
| **Gender, male %** | 126 | 70 | 144 | 60 |
| **Age, years** | 126 | 55.5 (12.4) | 144 | 61.3 (11.5) |
| **Duration of diabetes, years** | 125 | 2.7 (3.4) | 144 | 4.6 (4.9) |
| **Body weight, kg** | 122 | 68.4 (14.4) | 138 | 63.1 (12.7) |
| **BMI, kg/m^2^** | 118 | 25.5 (4.4) | 137 | 24.7 (4.1) |
| **HbA1c, %** | 124 | 9.6 (1.7) | 142 | 9.3 (1.7) |

BMI, body mass index; HbA1c, glycated hemoglobin; s.d., standard deviation
